# Congenital and Perinatal Viral Infections: Consequences for the Mother and Fetus

**DOI:** 10.3390/v16111698

**Published:** 2024-10-30

**Authors:** Mariam Al Beloushi, Huda Saleh, Badreldeen Ahmed, Justin C. Konje

**Affiliations:** 1 Women’s Wellness and Research Centre Hamad Medical Corporation, Doha P.O. Box 3050, Qatar; dr.mariamqa@yahoo.com (M.A.B.); hsaleh1@hamad.qa (H.S.); 2 Department of Obstetrics and Gynaecology, Qatar University, Doha P.O. Box 2713, Qatar; profbadreldeen@hotmail.com; 3 Feto Maternal Centre, Al Markhiya Doha, Doha P.O. Box 34181, Qatar; 4 Department of Obstetrics and Gynaecology Weill Cornell Medicine, Doha P.O. Box 24144, Qatar; 5 Department of Health Sciences, University of Leicester, P.O. Box 7717, Leicester LE2 7LX, UK

**Keywords:** congenital and perinatal infections, vertical transmission, cytomegalovirus, hepatitis virus, herpes simplex type-2 virus, parvovirus B19 virus, rubella virus, varicella and zika virus

## Abstract

Viruses are the most common congenital infections in humans and an important cause of foetal malformations, neonatal morbidity, and mortality. The effects of these infections, which are transmitted in utero (transplacentally), during childbirth or in the puerperium depend on the timing of the infections. These vary from miscarriages (usually with infections in very early pregnancy), congenital malformations (when the infections occur during organogenesis) and morbidity (with infections occurring late in pregnancy, during childbirth or after delivery). The most common of these viruses are cytomegalovirus, hepatitis, herpes simplex type-2, parvovirus B19, rubella, varicella zoster and zika viruses. There are currently very few efficacious antiviral agents licensed for use in pregnancy. For most of these infections, therefore, prevention is mainly by vaccination (where there is a vaccine). The administration of immunoglobulins to those exposed to the virus to offer passive immunity or appropriate measures to avoid being infected would be options to minimise the infections and their consequences. In this review, we discuss some of the congenital and perinatal infections and their consequences on both the mother and fetus and their management focusing mainly on prevention.

## 1. Introduction

**Figure 1:**
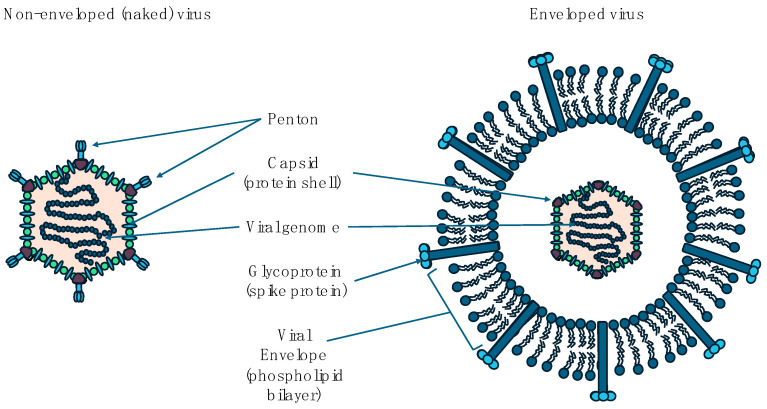
Cartoon of typical enveloped and non-enveloped viruses. Note that these do not reflect all the different forms of DNA/RNA (which maybe Double/single strained, circular linear etc.

Infections are common in pregnancy, but most have no effect on either the mother or the fetus. Where these have consequences, some can be devastating not only for the mother but also for the fetus if infected. Micro-organisms responsible for these infections include viruses, bacteria, protozoa, and fungi [1,2]. Viruses are the most common of these micro-organisms. They are small obligate intracellular parasites which can either be enveloped or non-enveloped. Structurally they consist of nucleic acid and proteins which make up a complete virus particle known as the virion. The nucleic acid which is either RNA or DNA is usually in the centre, surrounded by a protein coat called the capsid. The combined nucleic acid and this protein coat is referred to as the nucleocapsid. Although some viruses may only consist of this nucleic acid and protein coat (non-enveloped viruses), others have an additional membrane envelope surrounding the nucleocapsid (enveloped viruses). On the surface of some of these enveloped viruses are protein spikes that help attach the virus to the host. Figure 1 shows the differences between enveloped and non-enveloped viruses.

**Figure 2:**
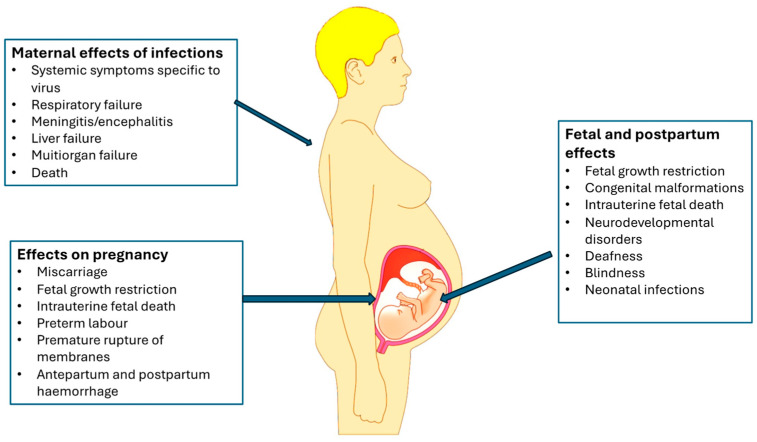
Overview of the effects of viral infections in pregnancy on the mother and fetus/neonate.

The effects of viral infections on the mother vary from none to severe consequences including maternal death. The most common severe effects include manifestation of generalised inflammation, respiratory failure, encephalitis, and multiorgan failure. The fetal consequences of viral infections depend on the gestational age at which the fetus is infected. In early pregnancy (especially in the early first trimester), infections that affect the fetus may either cause miscarriages or act as teratogens causing a variety of congenital malformations [3]. Some infections acquired in-utero may not cause congenital malformations but may cause significant neonatal morbidity. Most infections acquired in late pregnancy have a benign course, but some may cause significant morbidity and sometimes mortality in the neonatal and childhood periods. For those acquired intrapartum from cervico-vaginal secretions or postpartum, the onset of symptoms is related to the timing of the acquisition of the viruses as well as its incubation period (for example, the symptoms may not manifest for a few days or even weeks after delivery). The spectrum of the consequences of infections therefore varies from no effect to miscarriage to congenital malformations and fetal growth restriction to postpartum sequelae (which include symptoms of the infections) [1,2]. Figure 2 shows an overview of the effects of viral infections in pregnancy.

**Figure 3:**
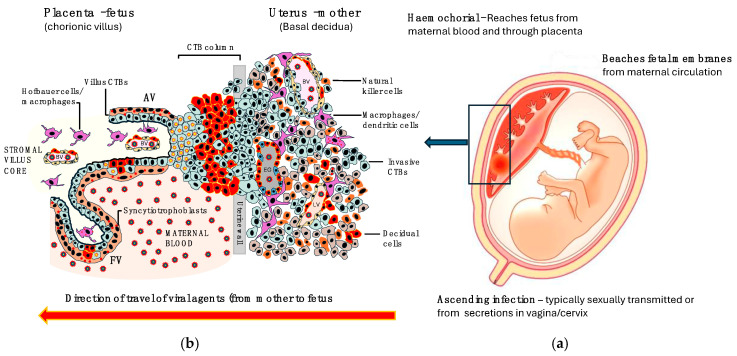
(**a**) Right shows routes of viral transmission to the fetus and (**b**) left shows how some viruses may reach the fetus. Some may infect the maternal decidual cells and then invade cytotrophoblast (CTB) cell columns and then traverse into the villi across fetal blood vessel into the fetal circulation or attach to fetal Hofbauer cells in the villous stroma. Others may traverse these barriers into the fetal circulation without infected the cells, The precise mechanism for most of these is not well understood. Abbreviations: CTB: cytotrophoblast; AV: anchoring villi; BV: blood vessel; EG: endometrial gland; FV: floating villi; LV: lymphatic vessel.

Vertical transmission (from the mother to the fetus) occurs through one of three ways: transplacental, intrapartum, or through breastfeeding. While the exact mechanism of transplacental transfer for most of these infections is unknown, the localisation/identification of the RNA or virion particles in the placenta/trophoblast from pregnant women infected with viruses indicates that these viruses do indeed get to the placenta prior to reaching the fetus [4]. For an infective organism to reach the fetus, it must breach protective barriers, some of which are physical, and others are immunological. The syncytiotrophoblast is the foremost barrier against haemochorial spread of infections and, once breached, the pathogen must then overcome the barriers within the villous stroma, which include fetus-derived macrophages or Hofbauer cells and the fetal microvasculature [5]. For some viruses, primary replication occurs in the decidual cells or in the cytotrophoblast which then forms a reservoir for the viruses at the maternal–fetal interface. From these reservoirs, the viruses invade into the fetal circulation. Figure 3 summarises the possible route of passage of viruses from the mother to the fetus. For details see the review by Megli et al. [5].

An ideal approach to minimising the effects of these infections on both the mother and fetus would be through the administration of effective and safe anti-viral agents. Unfortunately, unavailability of effective agents (and especially those that can cross the placenta) and vaccines against most of these viruses makes this difficult. The best approach in most cases is therefore education on behavioural modifications that reduce the risk of acquiring the infection, minimisation of their consequences by administering immunoglobulins (if available), or vaccinating susceptible individuals where this is available. In this review, we provide an update on some congenital viral infections (mainly cytomegalovirus, hepatitis A, B, D, and E, herpes simplex type-2, parvovirus B19, rubella, varicella zoster and Zika viruses) and their consequences for the mother and fetus, and how these infections can be treated, or their consequences minimised. Severe acute respiratory coronavirus (SAR-CoV)-2 and Ebola viruses (EBV) have been excluded (although there is some evidence of vertical transmission with SAR-CoV-2, there are only few reported cases and, furthermore, the reported consequences on the fetus/newborn are minimal, while EBV is restricted mainly to West African sub-region). The review of each virus should be read in tandem with the summary provided in Table 1 and Table 2.

## 2. Cytomegalovirus

Cytomegalovirus (CMV) is a large double-stranded DNA enveloped virus that belongs to the *Herpesviridae* family of viruses. It is able to establish lifelong latency with periodic reactivations in infected individuals [6,7].

### 2.1. Epidemiology, Clinical Course and Transmission

Cytomegalovirus is one of the most common viral infections with an estimated global seroprevalence of 83% [8]. Of those infected with the virus, 86% are women in the reproductive age group. The seroprevalence rate varies from one WHO geographical zone to another—the highest prevalence (90%) is in the Middle East and North Africa (MENA) Region, while the lowest (66%) is in Europe [9]. Infections with CMV can be in one of three forms: primary (individual has previously not been infected, i.e., is infected with the virus for the first time), latent, or reactivated latent. In a good number of cases, the reactivated latent infection may be with an entirely new strain of the virus [8].

Infections with CMV in most cases are subclinical or asymptomatic [10]. It has been suggested that in these individuals, the immune system is activated to ensure that viral replication is kept under control, but in a small number of cases replication is significant enough to cause symptoms. These are more severe in those who are immunocompromised (for example, those with HIV or on immunosuppressives drugs for organ transplant). Pregnant women, by virtue of physiological and immunological changes, are also slightly immunocompromised and may therefore manifest more severe symptoms compared to their non-pregnant counterparts [10].

Clinical features of CMV infection vary from generalised ‘flu-like’ symptoms (fever, muscle aches (myalgia), tiredness, a skin rash, nausea and a sore throat) to those of ‘glandular fever’ (e.g., fever, sore throat, swollen glands, abdominal pain, muscle aches and, in rare cases, jaundice) [10,11].

Transmission of the virus is by direct contact with infectious body fluids which include saliva, urine, tears, blood, semen, and breastmilk. The virus can also be transmitted sexually and through transplanted organs including blood [10,11].

### 2.2. Cytomegalovirus and Pregnancy

CMV infection is the most common viral infection in pregnancy that causes a plethora of congenital malformations with resulting disabilities [12]. It has been estimated that about 0.5% to 1.0% of newborns are infected with CMV [13,14]. Rates of previous infection vary; for example, in the USA, the overall CMV seroprevalence rate is ∼58% and this increases strongly with age. High CMV IgM titres are a strong predictor of low IgG avidity [15]—the most reliable serologic indicator of primary infection [16,17,18].

The virus reaches the fetus by invading and breaching the utero–placental barrier. Transmission to the fetus varies with gestational age; with the greatest transmission occurring in the third trimester. However, it is transmission in the first trimester that is associated with the highest risk to the fetus. Congenital malformations (which result mainly from infection in the first trimester), correlate with gestational age at seroconversion and placental pathology. Reported transmission rates are 30%, 38%, and 72% in the first, second, and third trimesters, respectively [19]. Primary maternal infection in the first trimester poses a 40% risk of vertical transmission, with 25% of infected fetuses having malformations. The malformations associated with CMV include cognitive dysfunction, seizures, sensorineural hearing loss, learning disorders, and microcephaly [19]. Hearing loss may be a delayed manifestation in up to about 7% of cases [20]. At birth, about 10% of infected newborns will have severe manifestations and about 23% will have at least one minor manifestation. A proportion will be asymptomatic with the symptoms (such as deafness) developing much later [21].

Unlike varicella zoster virus infection, which confers a lifelong immunity in most cases, previous infection with CMV does not. It does, however, modify the course of repeat infections such that the consequences (fetal and maternal) are less severe when compared to those from a primary infection. For example, women with preconception immunity to CMV have a low risk of vertical virus transmission (0.2% to 2.0%) [16]. Reinfection in a highly seropositive population tends to be with a different strain of the virus from the one that caused the primary infection that led to seropositivity [22].

A diagnosis of CMV infection in the mother cannot be reliably made based on seroconversion from negative to positive. This is because IgG and IgM antibodies persist for months after the primary infection making it difficult to time the infection with relation to the period of organogenesis. Avidity testing is therefore used to gauge the approximate time of infection especially if the timing is required to relate malformations to a possible infection that occurred before 20 weeks. Confirmation of intrauterine infection is made from the isolation of viral DNA in amniotic fluid by PCR, performed after 21 weeks of gestation. This is best performed about 7 weeks after maternal seroconversion, as testing prior to this may result in false negatives [21,23]. Detection of CMV DNA in urine and/or saliva at delivery is the best marker of newborns at risk for hearing loss [24].

Fetal growth restriction (FGR) is a consequence of congenital CMV early in pregnancy. The precise mechanism for this is uncertain but it is likely to include placental invasion with viral infection of trophoblasts and fetal membranes as evidenced from histopathological examinations that show changes consistent with inflammation and placentitis [23,25,26,27,28,29,30]. CMV replicates in the cytotrophoblasts and in the chorion and epithelial cells in the amnion [31,32]. The cytotrophoblast cells infected with CMV produce cmv IL-10 which reduces metalloproteinase-9 (MMP-9) levels and activity, thereby impairing invasion [33,34]. The infected trophoblast progenitor cells (TBPC) in the chorion in turn interfere with the growth of syncytiotrophoblasts (STBs) and chorionic villi [35]. A consequence of the loss of the TBPC reserve is a reduction in the responsiveness of the placenta to hypoxia, thus limiting the development of STBs and chorionic villi [36]. These changes affect placental development and predispose the fetus to growth restriction at early onset.

There is some evidence that pathological findings in the placenta vary with the gestational age at infection (being more severe with earlier infections) reflecting the greater risk of foetal growth restriction (FGR) with earlier infections. Placental pathology associated with congenital CMV infection diagnosed at mid gestation include pronounced villous maldevelopment, diffuse villitis, cytomegalic cells, and areas of necrosis and calcification [36]. Examination of placentas from women with CMV infection in late gestation has shown infected blood vessels (BVs) in the villus core, and villus necrosis and infected endothelial cells in the villus stroma. Interestingly these findings were without pathologic changes [37]. Cytotrophoblast, endothelial cells, and stromal fibroblasts of chorionic villi from placentas show viral proteins as evidence of CMV placentitis [38]. These findings suggest that CMV infection contributes to the FGR. In a study of paired maternal and cord sera and placentas from 19 pregnancies in which 5 of 7 cases were FGR due to primary or recurrent CMV infection, Pereira et al. showed large fibrinoids with avascular villi, oedema, and inflammatory CMV proteins in epithelial cells of amniotic membranes [35]. These findings provide evidence that congenital CMV infection should be considered as an underlying cause of FGR especially of the early onset type [35,39].

The factors responsible for transmission and severity of congenital CMV infection are not well understood [40]. Previous infection does not confer lifelong immunity, unlike that for rubella and varicella zoster (VZV). Thus, while congenital CMV infections have severe consequences in cases of primary infection, these have also been shown (though to much less extent and severity) in children born to mothers who have had a CMV infection before pregnancy (nonprimary infection) [40,41,42,43,44]. Previous infection, it would seem provides significant protection against intrauterine transmission; however, this protection is incomplete [45]. The prevalence of a congenital CMV infection at birth is directly related to maternal seroprevalence rates; these are highest in populations with higher CMV seronegative prevalence in women of reproductive age [46]. The rate of transplacental transmission of CMV decreases from about ‘25–40%’ in mothers with primary infection during pregnancy to <2% in those with pre-existing seroimmunity [19]. Why maternal immunity does not completely prevent reinfection (i.e., it is not sterilising and does not completely prevent reinfection) and thus vertical transmission is not well understood, but it has been suggested that reinfection with a different strain of CMV can lead to intrauterine transmission and symptomatic congenital infection [47]. Furthermore, accumulated data, especially from studies in highly seropositive populations, suggest that once intrauterine transmission occurs, pre-existing maternal immunity may not modify the severity of fetal infection and the frequency of long-term sequelae [41,42,43,48,49].

Most transmission occurs at birth with approximately 50% of infants born to mothers shedding CMV from the cervix or vagina at the time of delivery being infected [50]. Factors that increase lower genital tract shedding and therefore a greater risk of vertical transmission at birth include concomitant infection with other STIs, number of sexual partners (which is associated with a greater risk of STIs), and being of a younger age [51].

Following maternal infection, the virus is shed in body fluids including breastmilk, saliva, and tears. The virus has been detected in breastmilk in 13% to 50% of lactating women tested with conventional virus isolation techniques [52]. With the more sensitive polymerase chain reaction (PCR) technology, the presence of CMV DNA in breastmilk has been demonstrated in more than 90% of seropositive women [53]. Risk factors for postnatal transmission from milk have been shown to include an early appearance of viral DNA in milk whey, and a higher viral load in breastmilk [53]. Interestingly, while freeze-storing or pasteurization of maternal breastmilk has been shown to reduce the viral load, transmission of CMV to infants that have received treated breastmilk has not been documented [54].

### 2.3. Prevention and Treatment

Antiviral drugs licensed against CMV infection in immunocompromised (non-pregnant) women include ganciclovir, valganciclovir, cidofovir, foscarnet, and valaciclovir. Except for valaciclovir, the teratogenic and toxic effects of the others preclude their use in pregnancy. Jacquemard et al. [55] and Lereuz-Ville et al. [56] investigated the use of valaciclovir in pregnancies with CMV-infected symptomatic fetuses. Valaciclovir, a prodrug that is converted in vivo by esterases into the active drug aciclovir in the liver during first pass metabolism, was preferred because it has greater oral bioavailability than aciclovir (55% versus 10% to 20%) [57,58]. Aciclovir, however, has an excellent safety profile in pregnancy as it is not genotoxic in vitro, and there is considerable evidence that its use in the first trimester in humans is not associated with any increase in the rate of congenital malformations [59,60]. Both agents (aciclovir and valaciclovir) though have limited antiviral activity against CMV. In a series of 20 cases with CMV in pregnancy, Jacquemard et al. [55] offered oral valaciclovir for 7 weeks (range 1–12 weeks) at a dose of 8 mg/day for 7 weeks starting at 22–34 weeks (average 28 weeks). Of the 20 cases, 7 were terminations, 6 of which had evidence of progressive disease, and 1 termination was performed on parental request; 13 were live births (10 with a normal clinical examination at 6 months—follow-up was for 6–39 months), 2 had isolated unilateral sensorineural hearing loss (SNHL) and 1 had hearing loss, microcephaly, and incontinentia pigmenti). When these outcomes were compared to those of 24 untreated symptomatic CMV-infected fetuses, 14 (58%) in the untreated group were either terminated (n = 10), intrauterine foetal death (n = 1), or severe neonatal infection (n = 1). Ten (41%) infants were healthy compared to 71% in the treated group that did not undergo a termination. Further evidence of benefit for valaciclovir was generated from the “In Utero Treatment of Cytomegalovirus Congenital Infection with Valaciclovir (CYMEVAL)” trial—a phase II open label study [56]. Oral valaciclovir 8 mg/day was given for a median of 89 days to women with a moderately-infected fetus, presenting with non-severe ultrasound features which included extracerebral ultrasound abnormalities and/or mild ultrasound brain abnormalities. Treatment resulted in a significantly greater proportion of neonates born asymptomatic in the treatment group (82% versus 43%) and there were no maternal or neonatal adverse effects reported. It does therefore seem that valaciclovir may be the treatment option for confirmed CMV infection during pregnancy but more robust evidence from randomised trials would be much welcomed.

As there is currently no licenced vaccine for CMV, the best way to reduce the risk of infection during pregnancy is through behaviour modification. Simple hygiene-based measures that have been shown to reduce the risk of CMV acquisition include handwashing after contact with urine or saliva, and avoiding sharing utensils, drinks, or food with young children. Such educational interventions have been shown to be more effective in pregnancy. For example, in a study by Alder et al. [61] of seronegative women with a child younger than 36 months who received preventative information in pregnancy, the seroconversion rate was 1.2% compared to 7.6% in a group of women who did not receive such advice (*p* < 0.001), providing evidence that risk reduction is possible.

Intrauterine infection (congenital CMV) should be confirmed at birth by viral PCR from either urine or oral swabs obtained within 3 weeks of birth. For symptomatic neonates with congenital CMV infection, postnatal valganciclovir/ganciclovir treatment should be considered and commenced within the first 4 weeks of life. There is evidence that this treatment can reduce or prevent progression of SNHL and improve long-term neurodevelopmental outcomes in some infants [11,62,63].

## 3. Viral Hepatitis in Pregnancy

Acute viral hepatitis is caused by hepatitis A, B, C, D, and E viruses. The mode of transmission of these viruses as well as the clinical consequences on both the mother and the fetus/neonate vary.

## 4. Hepatitis A Virus (HAV)

The hepatitis A virus (HAV) is a non-enveloped, single-strand positive sense RNA virus belonging to the *Hepatovirus* genus and the *Picornaviridae* family [64]. There are six genotypes but only genotypes I to III infect humans [65,66]. Two infectious forms exist in the host—namely the naked virions which are shed in the faeces (hence transmitted faeco-orally) and the quasi-enveloped virions which circulate in blood [65,67].

### 4.1. Epidemiology Clinical Course and Transmission

Hepatitis A virus (HAV) is endemic in countries with poor hygiene and sanitation systems (predominantly in the Middle East, North Africa, Sub-Saharan Africa, South and Central Asia, and Latin America) [64]. It is a common cause of mainly self-limiting acute viral hepatitis with reported mortality rates from 0.3% to 0.6% [65,68]. World-wide, it is estimated that about 1.5 million new cases are reported annually. This is likely to be underreporting, however, as the true incidence may be much high since milder cases are likely to go unrecognised in view of its self-limiting nature [65,69,70].

HAV is transmitted via the faecal–oral route either by direct contact with an infected person or indirectly by the ingestion of contaminated water and/or food, especially raw and undercooked shellfish [71,72]. It has an incubation period of 28 days (range 15–50 days) [73]. Its prevalence is categorised as low, intermediate, or high based on the levels of anti-HAV-IgG in serum [62]. This categorisation is important as it provides guidance on the route of transmission and prevalence of anti-HAV-IgG. Transmission in high-endemic areas is mainly through contaminated water and, therefore, more than 90% of the populations would have anti-HAV IgG by the age of 10 years. Consequently, large epidemics are infrequent, as most people are immune from asymptomatic/mild HAV infection or acute hepatitis A during childhood [64,70,74,75,76]. In intermediate–endemic areas, on the other hand, most transmission is through ingestion of/drinking contaminated food and/or water, and the prevalence of anti-HAV IgG is equal to or >50% by age 30 years but <50% at the age of 15 years. Because of the wide distribution of anti-HAV-IgG, the virus causes large-scale cyclic outbreaks [77,78,79]. In low-endemic areas, transmission occurs mainly through food handlers, travel to high-endemic areas, or with oral–anal sex. The infection rates in these settings are very low, and <50% of people > 30 years have immunity against HAV [77].

Once the hepatitis A virus enters the body (from ingestion of contaminated food/water) it causes an intestinal infection. From there it spreads, probably by the bloodstream, to the liver, a target organ. Large viral particles are detectable in faeces from as early as 10–14 days after exposure and persist until peak elevation of serum aminotransferases, usually early in the acute phase of illness, but relatively infrequently after the onset of clinical jaundice. Clinical features vary from mild pyrexia to upper abdominal pain, and jaundice. Interestingly, the antibody to HAV that persists is also detectable late in the incubation period, coinciding approximately with the onset of biochemical evidence of liver damage. The virus produces pathological changes exclusively in the liver where it causes the following: cytologic necrosis, conspicuous focal activation of sinusoidal lining cells; accumulations of lymphocytes and more histiocytes in the parenchyma, often replacing hepatocytes lost by cytolytic necrosis predominantly in the periportal areas; occasional coagulative necrosis in the form of acidophilic bodies; and focal degeneration [80]. The virus itself is not directly cytopathic to hepatocytes, but liver injuries are mainly secondary to the host immune response. Viral clearance after primary infection is achieved by cellular immunity, whereas humoral immune response is responsible for protection and prevention of infection. Individuals with defects in cellular immune response, such as those with human immunodeficiency virus (HIV) infection, or on immunosuppressants following transplant can produce longer viral shedding with high infectivity, but without an apparent increase in the severity of symptoms [65].

### 4.2. HAV and Pregnancy

HAV is an uncommon infection in pregnancy and, when it occurs, vertical transmission is uncommon. However, there are numerous reports of vertical transmission with meconium peritonitis and perforation of the distal ileum—a very rare fetal complication of vertical transmission requiring surgery [81,82,83]. Although no serious outcomes associated with HAV infection in pregnancy have overall been reported [84], there are associations between acute infections and preterm labour, placental abruption, and premature rupture of fetal membranes, especially if the infection occurred in either the second or third trimesters [85]. Maternal pyrexia is likely to be the mechanism through which these complications are precipitated. It is extremely rare for infants born to mothers with HAV infection to be affected and indeed many tend to have normal antibody and transaminase levels. In the rare cases of mother-to-child HAV transmission, complications of fetal ascites, meconium peritonitis, neonatal icterus, and distal ileum perforation have been reported [85]. While it is a rare but accepted cause of mortality especially in those >50 years, there are no documented reports of mortality in pregnant women and infants exposed to HAV infection. In these cases, there is usually full resolution of the infection [86].

There is no evidence that the virus is transmitted through breastfeeding even though mothers infected with HAV have anti-HAV antibodies and HAV RNA in their breast milk. Breastfeeding is therefore not contraindicated in these women [87]. Administering immunoglobulin or the inactivated vaccine to children of infected mothers is likely to offer protection from HAV infection [88]. There is also evidence that administering the HAV vaccine to children <2 years of age, induces seropositivity that could persist for at least 10 years regardless of the presence of maternal anti-HAV IgG [89].

Since maternal anti-HAV IgG antibodies cross the placenta to the baby and may persist well into the second year of life, depending on the level of HAV endemicity and the average anti-HAV IgG antibody levels in a given maternal population [87,90,91], the timing of vaccination is critical for efficient HAV vaccination in high-endemic areas as high levels of maternal anti-HAV IgG antibodies present in the first year of life may impede the vaccine response. On this basis, it has been recommended that in high endemic areas children <1 year should not be vaccinated [88,92,93].

### 4.3. Prevention and Treatment

There is no specific antiviral therapy for hepatitis A [66], however pre-exposure (by vaccination) and post-exposure prophylaxis (with immunoglobulin) are recommended to provide protection for unvaccinated pregnant or potentially pregnant individuals, especially those travelling to or working in countries with high or intermediate HAV endemicity [94]. When administered within 2 weeks of exposure, post-exposure prophylaxis (PEP) with the HAV vaccine or immunoglobulin (IG), prevents infection with HAV [95,96]. Advantages of the vaccine over the immunoglobulin as PEP include ease of administration, greater acceptability and availability, induction of active immunity, and longer duration of protection [97]. Counselling and educating pregnant women or those who could become pregnant travelling to highly HAV endemic areas will undoubtedly reduce the risk of HAV infection in pregnancy. Pregnant women and women of reproductive age need protection against HAV before visiting HAV-endemic countries or low-income countries with poor sanitation and hygienic standards [65].

Vaccination is with an inactivated virus which is considered safe during pregnancy. It is available both in the monovalent and in combination with hepatitis B virus. Two doses are usually administered. After 2 weeks of the first dose, about 70% of individuals develop protective levels of antibodies [98]. Therefore, giving the HAV vaccine immediately before travel will ensure adequate protection in most individuals, because the incubation period for HAV is 15 to 50 days. The levels of antibodies are likely to persist for at least 10 to 29 years or perhaps for life after receiving the second dose of the HAV vaccine [96].

## 5. Hepatitis B (HBV)

Hepatitis B virus (HBV), an enveloped virus, is a 42-nm particle member of the *hepadnaviridae* family [89]. There are at least 10 Hep B virus genotypes (A-J) with distinct geographical distributions [98,99].

### 5.1. Epidemiology, Clinical Course and Transmission

The WHO estimates that there are over 257 million people living with chronic HBV worldwide, with the highest prevalence in Sub-Saharan Africa, Southeast Asia, and the Eastern Mediterranean regions. The disease is maintained in these regions by either maternal–fetal transmission or child-to-child spread [80]. It is estimated that chronic HBV disease is responsible for 900,000 deaths per year, secondary to either liver cirrhosis or hepatocellular carcinoma [80]. Rates of chronic HBV are related to the age at acquisition, approaching 100% following HBV infection in the neonatal period, exceeding 70% in early childhood and <1% after acute infection in post-pubertal immunocompromised individuals [100,101]. The prevalence of maternal HBV infection varies, with the overall prevalence being highest in regions identified as high risk. In the USA, the prevalence in pregnant women was 8.5/100,000 deliveries between 1998 and 2011 [102].

Acute HBV hepatitis presents with a variety of symptoms which include jaundice, right upper quadrant pain, nausea, vomiting, anorexia, low-grade fever, and fatigue. It is characterised by the presence of HBV surface antigen (HBsAg) in blood. Biochemically, serum transaminases are significantly raised and may peak in their thousands. About 1% to 2% of adults with acute infection will progress to acute liver failure. HBV has a prolonged incubation period that varies from 4 weeks to 5 months. Symptoms and changes in liver biochemistry typically appear after this period. In general, resolution occurs within 2 months of the acute infection (as evidenced by the clearance of HBsAg), but the development of chronicity may result in persistent liver injury. Neonates who acquire the virus either at the time of birth or shortly after, have a high rate of chronicity and may indeed enter an immunotolerant phase with high levels of HBV DNA in their serum, which may persist for decades. Preventing vertical transmission is therefore of great importance.

### 5.2. HBV Infection and Pregnancy

Symptoms of acute HBV infection in pregnancy are not different from those outside pregnancy. In the first trimester, the symptoms of nausea and vomiting may be confused with those of pregnancy and hyperemesis gravidarum. Because of the altered immune status induced by pregnancy, there is an increased risk of flare in women with chronic inactive and non-replicative HBV. When this happens, there is typically an increase in HBV DNA and increased levels of transaminases. Acute/chronic HBV does not increase fetal complications; however, where there is associated liver cirrhosis or failure, maternal morbidity and mortality may be increased. The risk to the fetus is mainly at the time of birth where transmission is high. It is not known to be transmitted through breastfeeding; however, infected mothers should be educated on preventative measures.

### 5.3. Prevention and Treatment

There are two aims of instituting treatment of HBV in pregnancy. The first is a reduction of liver injury (in those with high HBV DNA and elevated liver enzymes) in those with acute hepatitis; the second is to reduce the risk of vertical transmission. In the first category the treatment will be long-term. It is important to note that the risk of transmission to the infant is linked to maternal blood HBV DNA level—higher levels are associated with increased risk. For example, it has been shown that up to 25% of newborns will acquire HBV if the mothers’ HBV viral titre exceeds 200,000 IU/mL [103]. Treatment with nucleoside/nucleotide-based medications given in the third trimester has been shown to significantly reduce vertical transmission [104]. Guidelines from the American Association for the Study of Liver Disease (AASLD) and the European Association for the Study of the Liver (EASL) recommend administration of anti-viral therapy to women with HBV DNA levels of >200,000 IU/mL [105,106]. The antiviral agents, lamivudine and tenofovir have been used in various studies and shown to be safe and effective in reducing vertical transmission. Most of the data on these drugs come from treatment of HIV positive women [107]. The risk of vertical transmission can also be reduced by HBV vaccination combined with hepatitis B immune globulin (HBIG).

Vaccination against HBV is with a vaccine containing a recombinant hepatitis B surface antigen; hence it is safe during pregnancy. This vaccine offers a high protective efficacy especially in those who are immunocompetent where a three-dose vaccine regimen has been reported to achieve 90% to 95% protective efficacy, defined as a serum titre of the anti-HBs antibody of 10 mIU/mL or greater. Universal screening for HBV in pregnancy is advocated and vaccination of non-immune women during pregnancy is recommended for those at a higher risk of HBV exposure.

## 6. Hepatitis E (HEV)

Hepatitis E virus (HEV) is a single-stranded positive-sense RNA virus in the family of *Hepeviridae*. The virions are non-enveloped spherical particles found in faeces but exist as quasi-enveloped particles circulating in blood. There are two types of HEV virus-like particles (VLPs) that have been reported and at least eight major genotypes of HEV that have been identified from geographically distinct locations [107,108,109,110,111,112,113]. Genotypes HEV1 and HEV2 are the main pathogenic types associated with infections in humans [108].

### 6.1. Epidemiology Clinical Course and Transmission

HEV is the most common cause of acute viral hepatitis especially in young adults in many low- and middle-income countries [114,115,116,117]. It is transmitted mainly by the faecal–oral route with an average incubation period of six weeks (range 2–6 weeks). The highest attack rates are found in young adults, and high mortality rates of up to 20% have been reported in women during pregnancy. Clinically, acute cases are indistinguishable from other causes of acute viral hepatitis. There are approximately 20 million HEV infections world-wide with an estimated 3.3 million symptomatic cases [118,119]. The mortality from HEV is estimated to be about 56,600 annually [119]. The sero-prevalence of anti-HEV IgG ranges from 10% to 47% in countries with poor hygienic conditions.

Most patients with acute viral hepatitis from HEV recover completely but mortality rates vary from 1% to 4% [120,121,122,123,124,125,126]. In pregnant women, it has been associated with spontaneous miscarriages, stillbirths, and a maternal mortality rate of 20% to 40% in some parts of the world, especially in the Indian Subcontinent [120,121,122,123,124,125,126]. HEV infection can progress to fulminant hepatic failure, chronic hepatitis, and extrahepatic neurological and renal diseases [127]. The transmission and distribution of HEV varies with the different genotypes. For example, genotypes 1 and 2 are transmitted faeco-orally (through ingestion of contaminated food and water) while genotypes 3 and 4 (endemic in Europe and North America and indeed other high-income countries) are transmitted by eating undercooked pork, deer, and wild boar [128,129,130,131,132,133] with a primary zoonotic reservoir in these animals. Genotypes 5 and 6 are mainly present in Japan and infect boars and potential humans. While genotypes 7 and 8 are predominantly in the Middle East and China infecting camels and humans [134,135,136,137,138,139]. Contamination of water supplies and/or food with faecal material, especially after natural disasters, heavy rains, or drought is responsible for HEV outbreaks in about 90% to 95% of cases [121].

Most patients are asymptomatic. When symptoms develop, they include low-grade fever, nausea, vomiting, and anorexia. About 40% of patients develop hepatitis-like symptoms such as jaundice, pruritus, dark urine, and pale stools. Those who are immunosuppressed, either from infections such as HIV or on immunosuppressive drugs for solid organ transplant, may fail to clear the virus after primary infection, leading to chronic HEV infection (i.e., lasting >6 months) [140]. A laboratory diagnosis of HEV infection is based on the detection of the HEV antigen, HEV RNA, and antibodies against HEV [137]. Anti-HEV IgM antibodies, which represent recent exposure and last for up to 4–5 months, can be detected during the acute phase of the illness. Anti-HEV IgG antibodies, on the other hand, represent remote exposure and can last more than 10 years.

### 6.2. Hepatitis E and Pregnancy

Unlike HAV and HBV, HEV infection in pregnancy is associated with a high mortality, although this is mainly in low- and middle-income countries. This is more so for infections in the third trimester. In a review of 930 cases from Asia and Africa, a mortality of 15.9% (range 6.9% to 31%) was reported [138]. The difference in mortality between high-income countries and low-income countries may be secondary to better medical care and that infections in high-income countries are with the much lower pathogenic HEV-3 and -4 genotypes, which have also not been reported to cause congenital infection.

Vertical transmission occurs antenatally and intrapartum with a reported rate of 30% to 100% [138,139,141,142,143,144,145]. A study from the United Arab Emirates, for example, reported a vertical transmission rate of 100% with a resulting significant perinatal morbidity and mortality [143]. Although the anti-HEV antibody and HEV-RNA have been shown to be present in the colostrum of HEV-infected mothers, breastfeeding is not thought to be associated with transmission [146]. Postpartum transmission, however, may occur through close contact of mothers with their infants, especially when the mother has HEV- caused acute viral hepatitis [147]. Complications of intrauterine infections include premature delivery, low birth weight, stillbirth, and death [138]. It has been estimated that HEV may be responsible for 2400–3000 stillbirths annually [143]. Vertical transmission in HEV IgM-positive mothers appears to be significantly correlated with viraemia and a viral load of 13,300 copies/mL or higher [143].

The mode of maternal–fetal vertical transmission remains unclear, but it has been suggested that immune complexes with non-neutralised HEV particles may facilitate neonatal crystallizable fragment receptor (FcRn)-mediated transport. HEV replicates in the placenta, and the virion structural protein ORF3 has been detected in decidual cells, STBs, and stromal cells of the villus core, suggesting a high viral load and extrahepatic sites of viral replication [147,148].

### 6.3. Prevention and Treatment

Although there is currently no effective anti-viral treatment for HEV, there is however a vaccine that was licensed (by China) in 2011 with the trade name of Hecolin^®^. This vaccine contains an antigen that is a truncated version of the capsid protein encoded by ORF2 [144]. Although the safety and efficacy of this vaccine have been demonstrated in a large-scale phase III clinical trial [145], this was not during pregnancy. However, since it is not a live attenuated vaccine, it is likely to be safe during pregnancy. It must, however, be used during pregnancy with caution, although preliminary data suggest it to be safe for both the mother and fetus [149]. This vaccine has not yet had regulatory approval in the USA and other high-income countries.

## 7. Herpes Simplex Virus Type 2 (HSV-2)

The herpes simplex type virus II (HSV-2) is a member of Herpesviruses. It is a double stranded DNA (dsDNA) enveloped virus which causes a variety of diseases including genital and, in rare cases, oral blisters [150].

## 8. Epidemiology, Clinical Course and Transmission

Herpes simplex virus type 2 (HSV-2) is a sexually transmitted infection whose prevalence world-wide varies, is influenced no doubt by sexual habits and the prevalence of other STIs such as HIV. In Europe, a mathematical model estimated that 10.7% of females and 5.3% of males aged 15–49 years were infected with the virus [151,152]. In a study by Alareeki et al. [153] the pooled seroprevalence in the general population to HSV-2 in Europe was 12.4% (but in men who had sex with men, this was 27.8% and even higher in those living with HIV and people in HIV discordant couples, at 46.0% (95% CI: 40.1–51.8%). In female sex workers the seroprevalence was 63.2%. In 2016, the WHO estimated that 491.5 million people were living with HSV type 2 infection, equivalent to 13.2% of the world’s population aged 15–49 years [154]. In Mexico, 8.5% of pregnant women were found to have been infected, of these, 38.1% had vaginal HSV-2 shedding. This prevalence was higher in younger women compared to adolescents (12.1% vs. 4.3%) [155].

With a high seroprevalence in women of reproductive age, group studies have estimated the rates in pregnancy to be high and these rates also vary world-wide. In the USA, for example, the prevalence of HSV-2 infection in pregnancy is about 15.9% [156]. In Iran, the estimated rate in pregnant women was 5.8% but this was much higher in those who had had a miscarriage (19.7%) [157]. Various studies have reported prevalences in pregnant women of 30% in Haiti [158], 8.7% in India [159], 32% in a region in Ethiopia, 51.1% in Port Harcourt, Nigeria [160], and 49.1% in Zimbabwe [161]. These studies were institutional and therefore unlikely to reflect community prevalence; all the same, these are high.

Clinical features of genital HSV-2 depend on the stage of the infection and whether it is a primary or recurrent infection. Those with a primary infection have lesions typically on the vulva, vagina, and cervix. These may be associated with fever, malaise, anorexia, and bilateral inguinal adenopathy. Where the urethral is involved, women may present with painful micturition and/or urinary retention. Urinary retention may also occur because of sacral radiculomyelitis which causes neuralgia [150,162]. Following a primary infection, complete healing of the lesions occurs within several weeks. Recurrent infection, which is also common, tends to present with milder symptoms, again with lesions on the vulva, vagina, and cervix. About a third of women with HSV-2 infection have no recurrence, a third have up to 3 per year and another third more than 3 per year [150,163]. Symptoms during pregnancy may be more florid than those in non-pregnant women, as pregnancy is associated with an altered immune status. Disseminated HSV-2, which is very rare, may present with features of encephalitis, disseminated skin lesions, hepatitis, or a combination of these. It is more common in pregnancy especially in immunocompromised women such as those with HIV. The maternal mortality with disseminated HSV-2 infection is high [162,163].

Transmission of HSV-2 is by contact with genital or anal surfaces, skin, sores, or fluids of an HSV-2 infected person during sex. It is important to remember that it is possible to transmit the infection even if the skin appears normal (i.e., there are no obvious lesions).

### 8.1. HSV-2 and Pregnancy

Genital HSV-2 infection in pregnancy is common. The virus can be transmitted to the fetus predominantly at birth, although there are case reports of transplacental transfer. The effect of the virus on the neonate is influenced to an extent by whether it is a primary or recurrent infection and on the timing of delivery relative to the onset of the infection. The rate of transmission is greatest (30% to 50%) for primary infections occurring within 6 weeks of delivery. This is primarily because of persistent viral shedding in vaginal and cervical secretions at this time and that maternal antibodies would not have been generated enough to cross the placenta and offer the baby passive immunity. Other factors which affect transmission include the duration of rupture of foetal membranes, the use of foetal scalp electrode, and the mode of delivery [163]. Transplacental transfer is rare but there have been case reports of skin, eyes and CNS disease, foetal growth restriction, and intrauterine foetal death suspected to be secondary to intrauterine infection. Because of neutralising antibodies that would have crossed the placenta to the fetus, recurrent HSV-2 infections are rarely associated with severe neonatal disease. These antibodies, however, do not prevent spread of the virus to the brain. More commonly, vertical transmission of HSV-2 in cases of recurrence often results in asymptomatic or unrecognised infections, although there are also reported forms of localised disease which include local CNS, skin, eye, mouth, and pharyngitis [162,163].

### 8.2. Prevention and Treatment

A diagnosis should be confirmed by viral polymerase chain reaction (PCR) testing. Since this is an STI, other STIs should be excluded. Women with a primary infection should be offered oral aciclovir 400 mg three times a day for 5 days. Intravenous administration should be considered in those with disseminated disease. Although aciclovir does not eliminate the virus, it reduces the severity and duration of shedding. Daily suppressive therapy with aciclovir 400 mg three times a day from 36 weeks of gestation in those diagnosed with the disease in the first or second trimesters has been shown to reduce the rate of vertical transmission. In those diagnosed in the third trimester, treatment should be continued until delivery. In the presence of lesions at term or for a primary infection diagnosed within 6 weeks of delivery, a caesarean section should be performed. There are no vaccines against HSV-2. Prevention must be directed at minimising exposure to the virus by using barrier contraception and using appropriate hygiene measures to avoid spreading the virus [162,163].

## 9. Parvovirus B19 (PB19V)

Parvovirus B19 (B19V) is a small icosahedral particle virus with a non-enveloped single-stranded linear DNA (ssDNA). It belongs to the family *Parvoviridae*, the subfamily *Parvovirinae*, the genus *Erythrovirus* and Human parvovirus B19 type species. There are three genotypes (genotypes 1–3)- type 1 being the most prevalent. The virus is highly species and cell-type specific and infects only human bone marrow erythroid progenitor cells [164,165,166,167,168,169].

### 9.1. Epidemiology, Clinical Course, and Transmission

The virus has a world-wide prevalence that increases gradually with age from 2% to 20% at <5 years and to 40% to 80% at >18 years [4]. Transmission of B19V is mainly by aerosols during the incubation phase, which is 3–21 days. Within a week, very high titres of >10^10^ genomes/mL blood are reached without apparent symptoms. This is followed by the onset of nonspecific symptoms, which include fever, malaise, headache, and myalgia. Viral DNA levels decrease with the onset of these non-specific symptoms, and, at the same time, there is development of protective antibodies. In some cases, the infection resolves without symptoms, irrespective of the peak level of viraemia. The viral DNA gradually disappears from the blood within months, although it may persist in tissues at low levels (<10^4^ genomes/mL) for longer. In some cases, a rash appears 2 weeks after the infection. Antibody titres peak during the development of the rash. The IgM antibody disappears within several months, whereas the IgG antibody which is mainly against the conformational VP2 epitope and VP1u may persist for life. Although quantification of IgM antibodies or IgG avidity against VP1u may distinguish recent from previous infection, this is rarely indicated in clinical obstetrics.

### 9.2. PB19V and Pregnancy

Susceptibility to parvovirus B19 varies world-wide in women of childbearing age. For example, it has been reported that 26% to 44% of women of childbearing age in Europe and Japan are not immune [4]. Transmission is usually from contact with infected young children [170]. A seroconversion rate of ∼1% has in general been reported but this may rise to 13% during epidemics which are thought to occur every 4–5 years. Viral PCR for DNA is the gold standard diagnostic test for maternal infection, and this is usually performed in combination with assays of either the IgM antibody or IgG avidity [171]. These tests can be performed soon after exposure [172]. The rate of vertical transmission in the first trimester in those in the acute phase of the infection, where the viral load is high, is about 24% to 39%. The strongly expressed globoside receptor on differentiating cytotrophoblasts cells support the fact that the virus invades the placenta. The virus targets progenitor erythroblasts and because erythropoiesis in the fetus is significantly greater, this results in a viral load in the fetus much higher than in the mother [173].

The consequences of fetal infection vary depending on gestation. When transmission occurs before 20 weeks of gestation, the risk of fetal hydrops and death is about 4% to 10% [174]. The hydrops results from severe anaemia and hyperdynamic cardiac failure with resulting cardiac deficiency, oedema, and fluid in various compartments—notably the chest and peritoneal cavity [171]. Fetal hydrops may resolve spontaneously when the fetal immune system develops, but untreated, severe hydrops is always fatal. Interestingly, as the levels of IgG antibodies increase in the fetal circulation, viral DNA levels decrease, and fetal anaemia becomes less severe, suggesting that transcytosed IgG reduces infection [175]. Parvovirus B19 is the only treatable cause of non-immune hydrops fetalis. Treatment is by intrauterine blood transfusion to correct the resulting anaemia. This allows time for the fetal immune system and/or maternal antibodies to control the infection [176]. Babies infected by parvovirus B19 are viral DNA positive at birth and develop an active antibody response to the infection [171].

### 9.3. Prevention and Treatment

Like most viruses, there is no known effective anti-viral treatment for parvovirus B19 infection. Hydroxyurea and cidofovir are, however, active against the virus in vitro but there are no clinical studies to confirm benefit. Intravenous IgG can, however, suppress viral replication in cases of persistent infection [4]. There is no available vaccine against this infection and screening of women before or during pregnancy is not routinely performed.

## 10. Rubella Virus (RV)

Rubella virus (RV) is an enveloped virus and a member of the *Matonaviridae* family belonging to the genus *Rubivirus* [177]. The infection was the first to be associated with teratogenicity following the observation, in 1941, that ocular malformations, notably congenital cataracts were common in babies delivered to women who had been infected with the virus [178]. The virus causes a mild exanthematic ‘measles-like’ disease that was first described by German physicians, leading to the term “German measles” [179].

### 10.1. Epidemiology, Clinical Course and Transmission

The introduction and widespread adoption of rubella immunisation has had a major impact on the incidence of rubella infection. It has almost been eradicated in some countries. Prior to the introduction of immunisation, the peak incidence was in children aged 5 to 9 years with the highest rates in late winter and early spring [180,181]. The virus affects children of both sexes equally but, in adults, it affects women more than men. Outbreaks (post immunisation) have been reported especially in older adolescents and young adults [181,182]. In endemic areas, the reported incidence is about 1.3/100,000 in the general population [180]. Although the Centers for Disease Control and Prevention declared that RV was eliminated in the United States in 2005, due mainly to childhood vaccination programs with the attenuated RV strains, [183], most of the world population has not comprehensively (now compounded more than ever with vaccine hesitancy fuelled by social medial) received the RV vaccine; hence, CRS (congenital rubella syndrome) remains a major public health problem in Africa and large parts of Asia.

The virus is spread through aerosols generated from infected persons with whom contacts are made. When acquired, it first attacks the upper respiratory tract and titres from pharyngeal swabs at this stage have been reported to reach 1 × 10^5^ TCD_50_/_mL_ (which is 50% infectious with each 0.1-mL secretion) [183]. The virus is then spread (by infected lymphocytes and alveolar macrophages) from the respiratory tract to the regional lymph nodes where it causes lymphadenopathy. From here, it spreads throughout the body with symptoms manifesting in the joints and the skin. Symptoms vary from those of a mild disease (which may not be recognised as the symptoms are mild) to those of fever, malaise, and a rash. By the time the rash appears, the virus is being secreted in lacrimal fluid, cervical secretions, and synovial fluid. The rash, which is characteristically erythematous, is distributed all over the body and typically disappears within a few days, but the lymphadenopathy and joint symptoms may last for weeks. The most severe forms present with encephalitis which has been shown to complicate about 1:6000 cases, and importantly 1:5 (20%) of these cases is fatal [4]. Neutralising antibodies produced in response to this infection are directed mostly against the E1 spike protein and, to a lesser extent, to the E2 spike [184].

### 10.2. Rubella Virus and Pregnancy

Vertical transmission is common in pregnant women infected with RV. Since this is associated with congenital malformations, the transplacental route is most definitely one through which the virus reaches the fetus. It has been suggested that prolonged viraemia or infected monocytes in maternal circulation disseminate the virus to the intervillous space or lymphovascular channels in basal decidua. On entering the placenta, the virus replicates and causes placentitis. The virus has been demonstrated in the cytotrophoblast, endothelial cells of the placentas, the amniotic epithelium, and cells in basal decidua [185]. When it reaches the fetus, it infects various cells causing significant cell damage with resultant consequences. These consequences depend largely on the gestational age. In the first trimester, especially in the first 8 weeks about 75% of fetuses are infected [179]. It causes spontaneous miscarriages in up to 20% of these cases, and most will have congenital rubella syndrome (CRS)—some of these fetuses will be miscarried [183,184].

The spectrum of congenital rubella syndrome (CRS) includes sensorineural deafness (∼80%), cataracts (∼50%), and congenital heart disease (>50%). Other common defects include glaucoma, retinopathy, mental retardation, thrombocytopenia, hepatosplenomegaly, and fetal growth restriction (FGR) [183,184].

In a significant number of cases fetal growth restriction results, secondary to infection of endothelial cells that (a) causes necrosis of BVs and avascular villi which affects transport functions, and (b) an overall reduction in cell numbers following their infection with the virus [4]. The proportion of infants infected and having consequences drops in the second and third trimesters. There are virtually no consequences of infection in the third trimester, although some of the neonates may develop late onset disease. The spectrum of late onset diseases that have been reported includes cardiac abnormalities (58%), psychomotor retardation (62%), and mental retardation (42%) [183]. A study by Toizumi et al. [186] of children with congenital rubella syndrome found late-onset sequelae (in 60% of cases), including sensory defects, developmental delay, and autism spectrum disorder.

When the infection occurs in a susceptible pregnant woman (i.e., someone with absent rubella antibodies—i.e., neither previously vaccinated nor infected) the fetal consequences will usually point to the likely timing of the infection. However, with later infections, it may be difficult. A low avidity test would help in the postnatal diagnosis of congenital rubella virus infection [187], especially as, months after infection, transplacental maternal antibodies would have waned.

### 10.3. Prevention and Treatment

Immunisation against the rubella virus is a very effective means of preventing infections during pregnancy. Vaccination with the MMR vaccine is recommended in childhood. It is a live attenuated vaccine. Women who are susceptible (seronegative) and planning pregnancy should be vaccinated and advised to avoid pregnancy for at least 4 weeks. Although this vaccine has not been reported to cause congenital malformations, a study showed that amongst 661 neonates born to mothers who erroneously received the vaccine, 3% had signs of RV infection, but none had CRS [188]. The proportion of seronegative women varies from country to country depending on the policy on childhood vaccination. In the USA, for example, nearly all women of childbearing age have been vaccinated; whereas, in China, which does not have universal childhood vaccination, 40% of women of childbearing age are seronegative [189]. Globally, an estimated 105,000 cases of CRS occurred in 2010, virtually unchanged from 1996 [190]. Although the WHO recommended RV vaccination worldwide in 2011, [191], this has not been universally implemented and vaccine hesitancy, which unfortunately is increasing, is also affecting uptake by parents. Nonimmune pregnant women who have recently been exposed to RV may achieve passive immunization with normal immune globulin if administered soon after exposure. There is no current known effective antiviral therapy for RV.

## 11. Varicella–Zoster Virus (VZV)

The varicella zoster virus (VZV) is a double-stranded DNA enveloped virus closely related to the herpes simplex viruses (HSV). It causes chickenpox (varicella) and shingles (zoster)—the former predominantly in children and young adults, the latter predominantly in adults and, in rare cases, in children [192,193]. It is a human alpha herpesvirus 3 (HHV3) and one of nine herpes viruses that can infect humans. It is also able to survive in the external environment for hours [194].

### 11.1. Epidemiology Clinical Course and Transmission

Chickenpox, the primary VZV infection is common in children, in most of whom it causes a mild disease. The prevalence of VZV infections varies within regions and countries. In most countries most adults would have had the infection by the age of 15 years. In the USA, for example, about 4 million cases are reported annually with 100–150 deaths and more than 100,000 hospital admissions. Following a primary infection, lifelong immunity is developed in most cases. In Europe and North America, up to 90% (80% to 98%) of the population would have had the infection before the age of 15 years. In low- and middle-income countries, there are no accurate figures for those with previous infections, but estimates vary from 50% to 90%. During pregnancy, it is estimated to affect about 3/1000 pregnancies [195,196]

VZV enters the host through the upper respiratory tract where it replicates in the mucosa. It then spreads to the tonsils and lymphoid tissues from where infected T cells transport the virus in the blood stream to the skin and peripheral nerves. It has an incubation period of 10–21 days (average of 14 days). The vesicular rash which typifies chickenpox, appears 10–21 days after the infection [197] and takes about 4 days to crust. This rash is widely distributed (on the face, the trunk, and shoulders) reflecting the spread of the virus. Although it is typically vesicular and fluid filled, it may also be filled with pus (if superinfected with bacteria) and/or rupture with scab formation.

During the primary phase of VZV infection, the virions gain access to the sensory nerve cell bodies in ganglia by retrograde axonal transport and there, latent infection is established [4]. When replication of these latent infection is reactivated, the virus then spreads by anterograde axonal transport to reach the skin and manifest as shingles (zoster). This is typically a vesicular rash in the dermatome innervated by the affected ganglion. It may also occur in the eyes [198]. All the skin lesions (in cases of primary infection or reactivation in the form of shingles) contain high concentrations of infectious virus which can be transmitted to susceptible individuals. Spread of the virus is mainly through significant contact with an infectious individual. The period of infectivity is typically 24–48 h before the rash appears and until it crusts—about 4–5 days [195].

Clinical features of chickenpox include generalised malaise, severe headaches, backache, abdominal pain, nausea and vomiting, diarrhoea, a sudden onset of high fever, and a generalised rash which is characteristically maculopapular. In some cases (especially those who are unvaccinated) the infection may progress to become very severe presenting with central nervous system disease features (such as those of meningitis) bleeding, hypotension, multi-organ failure, and even death. For those with latent disease that becomes activated presenting as Shingles, the features will vary depending on the location (e.g., specific single or multiple dermatomes and may be contiguous or non-contiguous). These include dermatome rashes (maculopapular and vesiculate), pain along the nerve dermatome, facial palsy, pain in the ear and face, conjunctivitis and, in rare cases, blindness [196].

Transmission of VZV is by direct and significant contact with a VZV infected person. A significant contact is defined as being in the same room for at least 15 min. During this time, contact may be made with inhalation of aerosols from vesicular fluid of lesions on the skin, or droplets generated during the period of infectivity [195].

### 11.2. Varicella-Zoster Virus and Pregnancy

Although the mechanism by which VZV transmission from the mother to fetus in utero is unknown, vertical transmission occurs during pregnancy, at delivery and postpartum. Evidence for intrauterine infection comes from the isolation of VZV DNA in placentas and amniotic fluid, detection of viral replication proteins in infected cytotrophoblasts and fetal tissues [196,199], and the presence of VZV-specific IgM and IgG at birth in infants with clinical symptoms [200]. The mechanisms of virus dissemination identified for primary infection suggest VZV-infected T cells traffic to basal decidua, where the virus replicates and then spreads to the adjacent intervillous blood space in the placenta [4].

VZV is teratogenic and the gestational age at the time of infection has a significant bearing on the spectrum of malformations that result. These malformations are collectively referred as the “congenital varicella syndrome” (CVS). Infection in the first trimester results in VZV transfer across the placenta in 24% of cases, with 50% of the infected babies being symptomatic, whereas second- and third-trimester infections are rarely associated with congenital malformations [200]. Pregnant women with zoster (shingles) have not been shown to have fetuses with CVS or indeed other abnormalities. This is thought to be because these women already have IgG antibodies which cross the placenta and provide passive immunity to the fetus. VZV infection is not considered life threatening in most cases, but during pregnancy, because of the relative immune suppression and the impact of the gravid uterus on the respiratory system, VZV infection (especially respiratory) may be life-threatening. It may also result in spontaneous miscarriage, preterm delivery, or stillbirth.

### 11.3. Prevention and Treatment

Treatment with varicella–zoster immune globulin along with antiviral drugs (e.g., aciclovir) has been recommended for women exposed to or infected with the virus, irrespective of gestational age. Although aciclovir is not licensed for use in pregnancy, there are no reported teratogenic effects from a Danish Registry of >100,000 cases treated with famciclovir and aciclovir [201]. Aciclovir is recommended if the infected pregnant woman presents within 24 h of developing the rash [202,203]. This is given orally but, if there is evidence of a severe systemic disease, parenteral treatment would be recommended. Susceptible women who have had significant contact but have not developed the symptoms should be given the VZV immunoglobulin. Infants of mothers who develop the rash a week before delivery should also be given VZV immunoglobulin. The VZV vaccine is available and has been shown to reduce the frequency of infections. It is a live attenuated vaccine, hence should be avoided in pregnancy, although no congenital malformations have been reported in women who have been accidentally vaccinated.

## 12. Zika Virus (ZIKV)

The zika virus is a single-strand positive-sense enveloped ribonucleic acid (RNA) virus that belongs to the family, *Flaviviridae*. Members of this genus include dengue virus (DENV), yellow fever virus (YFV), West Nile virus (WNV), Japanese encephalitis virus (JEV), and tick-borne encephalitis virus. It was first isolated in the Zika forest of Uganda in 1947. A few reported cases of human infection with ZIKV in Africa and analysis of seroprevalence indicates that the virus had circulated silently for two decades [204]. The first large outbreak outside of Africa and Asia was reported on the island of Yap in Micronesia [205], and in 2013–2014, a second outbreak occurred in French Polynesia [206]. Thereafter, it gradually spread to New Caledonia, the Cook Islands, and Easter Island in the South Pacific [207]. A phylogenetic analysis of nucleotide sequences of isolates collected between 1947 and 2010 to determine the viral lineages revealed two main types—an African and an Asian type. The strain responsible for the Micronesia epidemic and Cambodian cases originated in Southeast Asia [208]. The virus was first reported in Brazil in 2014 from where it spread rapidly within South America [209,210]. It was not previously considered a major teratogen until a series of severe congenital malformations were reported in the Americas between 2015 and 2016 that were attributed to ZIKV [211,212].

### 12.1. Epidemiology, Clinical Course and Transmission

Although ZIKV was first reported in Uganda it has since spread to several countries. The exact prevalence is unknown but, in December 2021, the WHO stated that there had been documented evidence of the virus in a total of 89 countries [213]. The lack of accurate epidemiological data is related to the fact the in most cases the infection is asymptomatic; hence, unlikely to be diagnosed.

Most cases of ZIKV infections are asymptomatic and even when there are symptoms, they are non-specific. These include fever, arthralgia, myalgia, headaches, a maculopapular rash, and conjunctivitis. These mild symptoms may last for a few days to a week and rarely require hospitalisation. In a few cases it may cause Guillain-Barré syndrome [214].

Transmission is through the bite of an infected *Aedes aegypti* mosquito, although the *Aedes albopictus* mosquito has also been reported to transmit the virus. Interestingly, these mosquitoes, which breed in domestic water containers, are more likely to transmit the virus during the daytime than at night [215].

### 12.2. Zika Virus and Pregnancy

The epidemic in the Americas responsible for the outbreak of the various congenital malformations has been attributed to the Asian lineage ZIKV strains. This epidemic was first reported in northern Brazil causing microcephaly in utero and Guillain-Barré syndrome in adults [216,217]. Subsequently, prenatal maternal infection with ZIKV strains of the Asian lineage was associated with a plethora of malformations that collectively have been labelled ‘congenital Zika virus syndrome’ (ZVS). These include microcephaly, neurological impairment, cerebral calcifications, and retinal damage [218,219] (see Table for more details). While most transmissions are from the bite of the *Aedes aegypti* mosquito, sexual transmission of ZIKV has also been reported [220]. The magnitude of the intrauterine consequence of ZIKV infection stems from its link to more than 2700 cases of microcephaly among confirmed maternal infections in the Americas during recent epidemics [221]. RNA of the virus has been found in neonatal brain, the placenta, and amniotic fluid of affected babies, confirming transplacental transmission and therefore implicating ZIKV as aetiological in these malformations [222,223,224]. The reported rates of congenital malformations vary with gestational age at intrauterine infection. In a series of 2549 pregnancies from USA territories, with laboratory evidence of maternal infection from January 2016 to April 2017, in the first, second, and third trimesters, reported rates of congenital malformations rates were 8%, 5%, and 4%, respectively [225]. The rate of microcephaly reported in a series from Brazil varied from 0.03% to 17.1% depending on the geographical area [226]. It has been suggested that the wide variation in rates of congenital malformations could be secondary to possible modifiers such as undernutrition, dense population in urban slums, and lack of information on measures to prevent infection during pregnancy [227,228].

That the virus is responsible for a series of congenital malformations most of which occur at the time of organogenesis indicates that transplacental vertical transmission must occur early in pregnancy. The virus is known to target cells of the developing placenta and contribute to pathology. ZIKV has been shown to replicate in primary cells isolated from chorionic villi and amniochorionic membranes, including CTBs, Hofbauer cells, umbilical cord endothelial cells, TBPCs, and amniotic epithelial cells [229,230,231]. The virus also infects numerous cell types of basal decidual explants, targets proliferating CTBs in cell columns and villus sprouts and Hofbauer cells in stromal villus cores anchoring villi, but not STBs, possibly because of the antiviral effects of IFN-λ [229,232,233,234].

Several studies—mainly on first trimester villous explants have been undertaken to explain why American strains disseminate to the pregnant uterus and replicate in the placenta, while the African strains do not. Four possible explanations have been suggested from these studies. Firstly, the main targets of ZIKV infection in humans, CD14^+^CD16^+^ monocytes strongly produce the chemokine CXCL12/SDF-1 and the cytokine IL-6 [235]. These infected monocytes could cross uterine BVs into basal decidua where they differentiate into CD14^+^ macrophages that inhibit dNK cell functions [236]. Furthermore, ZIKV infection in endometrial glands, macrophage/dendritic cells, and decidual cells could increase virus titres in the intervillous blood space [230,232,237]. Secondly, the fact that the African prototype strain displayed more rapid replication kinetics and produced higher virus titres in human dendritic and placental cells, compared with the American (Nicaraguan) strains, may imply it stimulates a strong cellular immune response that may indeed suppress infection [221,229,237] and thus inhibit vertical transmission. The third explanation comes from analysis of villus explants infected with either the American or the African strains which showed differences in functions of infected CTBs [221]. Explants infected with the American strains replicated in proliferating cell columns and differentiated into infected CTBs that could invade basal decidua while explants infected with the African strains were severely impaired and thus had limited ability to invade the basal decidua. The fourth possible explanation is the ability of the American (Nicaraguan) strains to replicate in Hofbauer cells more frequently and cell proliferation suggested a source of infection that spreads to fetal BVs in villus cores compared to the African strain [226].

### 12.3. Prevention and Treatment

There are currently no antiviral agents or vaccines approved for use against Zika viruses. Developing a safe and immunogenic vaccine against the Zika virus remains an unmet medical need. One of the platforms that have reached clinical trial is the mRNA vaccine. Two randomised, placebo-controlled, dose-ranging, multicentre, phase 1 trials, one with mRNA-1325 (mRNA-1325 trial) and the other with mRNA-1893 (mRNA-1893 trial), have been performed. Three dose levels of mRNA-1325 (10, 25, and 100 μg) were used and these were generally well tolerated, but the vaccine elicited poor Zika virus-specific nAb responses after one dose. On day 57, all evaluated mRNA-1893 dose levels induced robust Zika virus-specific nAb responses, independent of flavivirus serostatus, that persisted until month 13. The conclusion from these findings was that the development of mRNA-1893 against Zika virus, which was well tolerated at all evaluated dose levels and induced strong Zika virus-specific serum nAb responses after two doses, regardless of baseline flavivirus serostatus should be continued [238].

## 13. Summary and Conclusions

Viral infections in pregnancy are common and the relatively immunocompromised status of the women, in some cases, is responsible for the more severe disease. Some of these viruses cross the placenta, and/or are secreted in various body fluids including vaginal and cervical secretions and breastmilk and infect the fetus/newborn. The consequences on the fetus/newborn depend on the virulence of the virus and the gestational age at infection. These effects include miscarriages, congenital malformations, preterm birth, stillbirth, and neonatal morbidity. Precisely how some of these viruses reach the fetus is uncertain, however the basal decidua has been shown to be invaded by most of the viruses that reach the fetus. Protection against these infections may be from maternally acquired antibodies (IgG from previous infections) or from immunisation. There are currently very few approved anti-viral agents for use in pregnancy and for most of these infections, prevention is the best approach for avoiding them. Table 1 and Table 2 summarise the viral infections that have been discussed, their incubation periods, mode of transmission, diagnostic approaches, consequences on the fetus and/or neonate, and treatment/prevention. Once diagnosed, a multidisciplinary approach to management must be adopted. This should include a foetal medicine physician, a virologist, and neonatologist where appropriate.

## Figures and Tables

**Table 1:** Incubation period, clinical features diagnosis, treatment and prevention in the mother.

Virus	Mode of Transmission to the Mother	Incubation Period	Clinical Features in the Mother	Diagnosis of Maternal Infection	Treatment/Prevention
CMV	Direct contact with infected body fluids—saliva, urine, tears, blood. Close contact with infected person	3–12 weeks	Asymptomatic, myalgia, headaches, fever, sore throat, tiredness, and skin rash, glandular enlargement and jaundice.	Serology combined with avidity test to help time infection.	No licensed anti-viral drug (but valaciclovir shown to be beneficial)
HAV	Faeco-oral	28 (15–50) days	Mild fever, upper abdominal pain, and jaundice	Serology	Post exposure prophylaxis with immunoglobulin and Vaccination
HBV	Direct contact with infected body fluids (blood, tears, semen, sweat, vaginal and cervical secretions). From sharing needles & sexually	90 (160–150) days	Fever, right upper abdominal pain, nausea, vomiting, jaundice, fatigue and generally feeling unwell	Serology (HBsAg & if positive, HbeAg) then Viral PCR to quantify HBV	Nucleoside or nucleotide analogues (lamivudine, telbivudine, or tenofovir) during the last trimester in highly viraemic mothers HBIG to mothers and neonate
HEV	Faeco-oral	6 (2–9) weeks	Asymptomatic, low-grade fever, nausea, vomiting and anorexia; pruritus, dark urine, pale stools, and jaundice	Serology	No recommended treatment Chinese vaccine available
HSV-2	Direct contact with genital or anal surface (with lesions), skin sores of fluid such as vaginal or cervical secretions	2–12 days	Mild fever, vulval pain, blisters or rash on vulva, vagina, painful micturition, urinary retention, malaise, anorexia, and bilateral inguinal adenopathy	Viral PCR of vesicular fluids	Oral aciclovir 400 mg three times per day for 5 days or IV if disseminated disease
PB19V	Airborne (contact with droplets)	3–21 days	Low grade fever, malaise, headaches, and myalgia	Serology (IgM or seroconversion). Note maternal IgG & IgM may persist for time after acute infection	No anti-viral agent Intrauterine transfusion corrects hydrops
RBV	Airborne (contact with droplets)	4–14 days	Mild fever, malaise, nausea, characteristic erythematous rash, lymphadenopathy and encephalitis.	Serology (Positive maternal IgM antibody or antibody, IgG seroconversion, or a ≥4-fold rise between acute and convalescent IgG titres).	No recommended anti-viral treatment. Vaccination available
VZV	Direct person-to-person contact and Airborne (contact with droplets)	10–21 days	High fever, malaise, muscle pain (myalgia), nausea, vomiting, severe headaches, backache, abdominal pain, diarrhoea and characteristic generalised maculopapular rash, meningitis, hypotension multiple-organ failure)	Serology	Aciclovir started within 24 h of the rash
ZIKV	Animal vector (Mosquito bite)	3–14 days	Asymptomatic, fever, arthralgia, myalgia, headaches, conjunctivitis and a maculopapular rash	Serology (Maternal IgM—detected from 4 days after infection [note may persist for 12 weeks after acute infection]) Avidity test will help time infection	No recommended anti-viral agent available

CMV: cytomegalovirus; HAV: hepatitis A virus; HBV: hepatitis B virus; HEV: hepatitis E virus; PB19V: parvovirus B19; RBV: rubella virus; ZIKV: zika virus; VZV: varicella virus.

**Table 2:** Risk of vertical transmission, fetal/neonatal consequences, diagnosis and treatment.

Virus	Risk of Vertical Transmission	Foetal/Neonatal Consequences	Diagnosis in Utero/after Delivery	Treatment of the Neonate
CMV	25% to 40% with primary infection and <2% with secondary infection	Microcephaly, foetal growth restriction, low birth weight, hepatosplenomegaly, sensorineural deafness, retinitis, thrombocytopenia, visual impairment	Amniocentesis for viral PCR ~5 weeks from infection. At birth, viral PCR from urine or oral swabs obtained within 3 weeks.	Valganciclovir or ganciclovir commenced within 4 weeks.
HAV	Very rare (few reported cases)	Ascites, meconium peritonitis, perforation terminal ileum, Jaundice	Not usually performed in utero Neonatal blood for serology and virology	No specific treatment
HBV	70% to 90% for hepatitis e antigen positive mothers and 20–40% for hepatitis e antigen negative	Persistent chronic hepatitis	Not usually performed in utero Neonatal blood for serology and virology	Intramuscular HBIG 0.5 mL to neonates and HBV vaccine within 12 h of birth
HEV	23% to 50%	Miscarriage, stillbirth, and neonatal hepatitis E infection	Not usually performed in utero Neonatal blood for serology and virology	No specific treatment
HSV-2	30% to 50% with primary infection and 0–3% with recurrent infection at the time of vaginal delivery	Neonatal herpes presenting with Skin, mouth and/eye disease; Local CNS (e.g., encephalitis), hepatitis, and disseminated infection	Not usually performed in utero Neonatal swabs (nasopharynx, anal, conjunctivae, mouth) for viral isolation/PCR	Aciclovir (intravenous) at a dose of 20 mg.kg every 8 h until active disease excluded
PB19V	Up to 33%	Hydrops fetalis, myocarditis	Viral PCR of cord blood	If anaemia, red cell transfusion. If high viral load persist—consider immunoglobulin (IVIG)
RBV	80% in the 1st trimester with up to 90% of fetuses affected. 25% to 30% affected >16 weeks with minimal effect of the fetus	Microcephaly, cataract, congenital glaucoma, congenital heart disease, hearing impairment, hepatosplenomegaly, purpura, jaundice, radiolucent bone disease developmental delay, pigmentary retinopathy	Viral isolation and RT-PCR form nasopharyngeal swabs	No specific treatment available
VZV	24% in 1st trimester	Affect skin, eyes and CNS and limbs. Eyes—chorioretinitis, cataract, nystagmus, cortical atrophy Limbs—atrophy, malformed digits, hypoplasia **CNS**—microcephaly, atrophy of the brain **Autonomic nervous dysfunction**—neurogenic bladder, hydronephrosis, oesophageal dilatation gastrointestinal reflex) Neonatal disease—pneumonia, meningoencephalitis, severe coagulopathy	From characteristic features of congenital VZV or detection of virus with PCR from neonate (blood or lesions)	Varicella immunoglobulin (VZIG) to neonate born within 5 days of the mother’s rash or 2 days after the rash develops. Not given to those whose mothers had shingles. Intravenous aciclovir to infected neonates especially those with disseminated disease
ZIKV	47% (26% to 76%)	Microcephaly, brain atrophy, cerebral and ocular calcifications, ventriculomegaly, periventricular cysts, callosal abnormalities, vermes agenesis, cerebellar atrophy, cortical atrophy	Classical features of congenital ZIKV syndrome; Swabs from placenta and cord blood for viral PCR. IgG and IgM in cord blood	No specific treatment

CMV: cytomegalovirus; HAV: hepatitis V virus; HBV: hepatitis B virus; HEV: hepatitis E virus; PB19V: parvovirus B19; RBV: rubella virus; ZIKV: zika virus; VZV: varicella virus; PCR: polymerase chain reaction; RT-PCR: reverse transcription PCR; CNS: central nervous system; HBIG: hepatitis B immunoglobulin; IVIG: intravenous immunoglobulin.
